# Rabbit Hemorrhagic Disease Virus Isolated from Diseased Alpine Musk Deer (*Moschus* *sifanicus*)

**DOI:** 10.3390/v12080897

**Published:** 2020-08-17

**Authors:** Shijun Bao, Kai An, Chunguo Liu, Xiaoyong Xing, Xiaoping Fu, Huiwen Xue, Fengqin Wen, Xijun He, Jingfei Wang

**Affiliations:** 1 College of Veterinary Medicine, Gansu Agricultural University, Lanzhou 730070, China; ankai1990826@163.com (K.A.); xingxiaoyong6123456@126.com (X.X.); fuxp@gsau.edu.cn (X.F.); xuehw@gsau.edu.cn (H.X.); FengQinW@163.com (F.W.); 2 State Key Laboratory of Veterinary Biotechnology, Harbin Veterinary Research Institute, Chinese Academy of Agricultural Sciences, Harbin 150069, China; liuchunguo@caas.cn (C.L.); hexijun@caas.cn (X.H.)

**Keywords:** rabbit hemorrhagic disease virus (RHDV), isolation, Alpine musk deer (*Moschussifanicus*), China

## Abstract

Rabbit hemorrhagic disease virus (RHDV) is the causative agent of rabbit hemorrhagic disease (RHD), and its infection results in mortality of 70–90% in farmed and wild rabbits. RHDV is thought to replicate strictly in rabbits. However, there are also reports showing that gene segments from the RHDV genome or antibodies against RHDV have been detected in other animals. Here, we report the detection and isolation of a RHDV from diseased Alpine musk deer (*Moschus* *sifanicus*). The clinical manifestations in those deer were sudden death without clinical signs and hemorrhage in the internal organs. To identify the potential causative agents of the disease, we used sequence independent single primer amplification (SISPA) to detect gene segments from viruses in the tissue samples collected from the dead deer. From the obtained sequences, we identified some gene fragments showing very high nucleotide sequence similarity with RHDV genome. Furthermore, we identified caliciviral particles using an electron microscope in the samples. The new virus was designated as RHDV GS/YZ. We then designed primers based on the genome sequence of an RHDV strain CD/China to amplify and sequence the whole genome of the virus. The genome of the virus was determined to be 7437 nucleotides in length, sharing the highest genome sequence identity of 98.7% with a Chinese rabbit strain HB. The virus was assigned to the G2 genotype of RHDVs according to the phylogenetic analyses based on both the full-length genome and VP60 gene sequences. Animal experiments showed that GS/YZ infection in rabbits resulted in the macroscopic and microscopic lesions similar to that caused by the other RHDVs. This is the first report of RHDV isolated from Alpine musk deer, and our findings extended the epidemiology and host range of RHDV.

## 1. Introduction

The Alpine musk deer (*Moschus sifanicus*) is a musk deer species belonging to the genus Moschus of the family *Moschidae* and is found only in the eastern Himalayas in Nepal, Bhutan, India, and China. In China, this animal is native to the plateau regions of several northwestern provinces, including Tibet, Qinghai, Sichuan, Xinjiang, and Gansu. The total number of this animal has been decreasing continuously worldwide; therefore, the International Union for Conservation has listed it as Endangered. The Alpine musk deer is facing a threat of extinction due to habitat destruction by deforestation and livestock farming, biological use as traditional medicine, and fatal diseases [1]. To protect this animal species, China has set up several national reserves, and one of them, Xinglongshan National Nature Reserve, is located in the Xinglong mountain, Gansu Province [2].

Rabbit hemorrhagic disease virus (RHDV), the causative agent of rabbit hemorrhagic disease (RHD), belongs to the *Lagovirus* genus in the *Caliciviridae* family. RHDV virions are nonenveloped spherical particles with a diameter between 30 and 40 nm [3]. The viral genome is a positive-sense single-stranded RNA, which is approximately 7437 nucleotides (nt) in length and is composed of two open reading frames (ORFs)—ORF1 and ORF2. ORF1 encodes the major capsid protein VP60 and several nonstructural proteins, including a helicase, a protease, and an RNA-dependent RNA polymerase (RdRp), while ORF2 encodes the minor structural protein VP10 [4]. The VP60 gene is always used to deduce the phylogenetic relationships among RHDV isolates.

The highly virulent RHDV was first reported in domestic rabbits in China in 1984 [5], from where the virus has spread to Europe and other continents [6,7,8,9,10,11,12]. RHDV infections result in high morbidity (100%) and mortality (70–90%) in adult European wild rabbits [13]. Historically, RHDVs have been divided into six genogroups (G1–G6) based on temporal distributions [14], and belong to a single serotype. RHDV2, a new variant of RHDV, emerged in France in 2010 and represents a unique branch in the phylogenetic tree [15]. Since then, RHDV2 viruses have been reported in many European countries [14,16,17,18] and other continents [19,20]. G1, G2, G6, and RHDV2 viruses have been reported in China, but the dominant circulating strains are from the G6 group [21,22]. RHDV is believed to have very strict host specificity, and there is no evidence to show that this virus can naturally or experimentally infect animals other than rabbits [23]. However, antibodies against RHDV have been detected in animals that live in sympatry with rabbit populations infected with RHDV [24], and RHDV RNA has also been isolated from sympatric wild animals, including rodents [25] and wild Tasmanian devils [26]. The possible roles of these animals in the ecology of RHD and whether RHDV can infect and cause diseases in other animal species remain unknown.

During December 2010 to January 2011, the Xinglongshan National Nature Reserve experienced acute fatalities in Alpine musk deer, resulting in the death of a total of 105 young animals (less than one year old) of both sexes, with a case-fatality rate of approximately 100%. Most of the animals died without visible clinical signs. The causative agent of this disease has not yet been determined. In this study, we report the identification of an RHDV strain from tissue samples collected from diseased Alpine musk deer, and we also compared the new isolate with previously characterized strains in terms of phylogeny and pathogenicity.

## 2. Materials and Methods

### 2.1. Ethics Statement

The animal experiment was approved by the Animal Management and Ethics Committee of Gansu Agricultural University (10 April 2015). This study was carried out in strict accordance with the recommendations in the Guide for the Care and Use of Laboratory Animals of the Ministry of Science and Technology of the People’s Republic of China (GB 14925-2001). Written informed consent was obtained from the relevant responsible person of Xinglongshan National Nature Reserve involved in our study.

### 2.2. Sample Collection Site

The diseased Alpine musk deer were raised in an enclosure in the Xinglongshan National Nature Reserve (E 103°50′–104°10′, N 35°38′–35°58′). A total of ~1000 deer were kept there at the end of 2010. These animals were fed with forage and occasionally leaves collected from their natural habitat.

### 2.3. Clinical, Histopathological and Immunohistochemical Examination

To investigate the potential cause of the disease, we conducted field investigations and postmortem examinations in January 2011. During the investigation, three dying Alpine musk deer at approximately 6 months of age were examined for clinical and postmortem signs. To examine their histological changes, tissue samples, including lung, liver, spleen, and kidney were collected and fixed in 10% neutral phosphate-buffered formalin, routinely processed and stained with H&E for microscopic examination. The tissue samples for virus isolation and bacterial culture were also collected and stored at −80 °C and 4 °C, respectively. In order to detect the RHDV antigen, immunohistochemistry was performed on livers of the dead Alpine musk deer. The primary antibody of the mouse anti RHDV VP60 protein was prepared by ourselves, with the working concentration of 1:100, and the secondary antibody of goat anti mouse IgG (H + L) conjugated with HRP (Invitrogen, CA, United States) was diluted at 1:5000. The immunohistochemistry was conducted as previously described [11].

### 2.4. Sequence Independent Single Primer Amplification (SISPA) and Sequencing

SISPA was conducted to identify potential viral agents in the tissue samples. In brief, a total of 5 g of the tissue mixture from each of the three animals was homogenized in 10 volumes of phosphate-buffered saline (PBS) and freeze-thawed repeatedly three times. The homogenates from the three animals were pooled and centrifuged at 10,000× *g* for 20 min to remove cell debris, filtered through a 0.22 μm syringe filter, and stored at −80 °C for further use. The pellet was resuspended in 1 mL PBS after ultracentrifugation at 160,000× *g* and 4 °C for 4 h (Beckman Coulter Optima XPN-100 Ultracentrifuge). To remove the exogenous nucleic acid contamination, the sample was treated with 10 U each DNase I and RNase I at 37 °C for 3 h. Viral DNA/RNA was extracted from samples with the Body Fluid Viral DNA/RNA Kit (Axygen, Wujiang, China) according to the manufacturer’s instructions. First-strand cDNA was synthesized with SuperScript I reverse transcriptase (Takara, Dalian, China) and K9N primers (Table 1) [27,28], and second-strand cDNA synthesis was then conducted by using Klenow fragment polymerase (Takara, Dalian, China). PCR amplification of viral nucleic acids was performed with LA *Taq* polymerase (Takara, Dalian, China) and K primers (Table 1). Fragments larger than 500 bp were purified by 1% agarose gel and subcloned into the pMD 18-T vector (Takara, Dalian, China) for sequencing by Comate Bioscience Co., Ltd. (Jilin, China).

### 2.5. Negative Staining Electron Microscopy (EM)

A total of 2 g of the liver was homogenized in 5 volumes of sterilized PBS. Large cell debris was removed by centrifugation twice at 4000× *g* for 20 min, and supernatants were collected and centrifuged at 10,000× *g* for 30 min. The pellets were resuspended in 100 µL PBS. A 25 µL drop of the sample suspension was adsorbed to a glow-discharged carbon-coated copper grid, washed with two drops of deionized water, and stained with 2.5% phosphotungstic acid for 30 s. The grids were examined by an electron microscope (Hitachi 7650) at an acceleration voltage of 80 kV.

### 2.6. Bacterial Culture and Virus Isolation

To identify potential pathogenic bacteria, tissue specimens were cultured in aerobic and anaerobic conditions on enrichment media (blood agar-OXOID) at 37 °C for 24 h. To isolate possible viral agents, tissue homogenates prepared for virus isolation were inoculated in Vero, Hela, MDBK, MDCK, CRFK, and RK-13 cells and blindly passaged for three generations. Cytopathic effect (CPE) was observed daily during the inoculation.

### 2.7. Genome Characterization

To obtain the full-length genome sequence of the RHDV detected in this study, a set of primers (Table 1) was designed based on the reference genome of the RHDV CD/China strain (AY523410.1), and one-step RT-PCR was carried out to amplify each gene fragment. The amplicons were purified using a TIANgel Midi Purification Kit (TIANGEN, Beijing, China), subcloned into the pMD18-T vector, and sequenced by Genewiz Biotech Co., Ltd. (South Plainfield, NJ, USA). The genomic sequences were assembled using the SeqMan program implemented in Lasergene 7.1 [29]. The subsequent multiple alignment of the assembled full-length genomes was performed with Clustal W [30]. The complete genome of RHDV GS/YZ was deposited in GenBank under the accession number MN478485.

To analyze the phylogeny of the new RHDV isolate, we constructed phylogenetic trees based on the nucleotide sequences of the full-length genome and VP60 of the new isolate and 85 reference RHDV strains downloaded from GenBank. Multiple sequence alignments were completed with Clustal W, and then, the aligned results were submitted to build neighbor-joining trees using the Kimura 2-parameter evolutionary model, implemented in MEGA 7 [30]. Bootstrap resampling was performed on 1000 replicates.

### 2.8. Animal Experiment

A total of nine New Zealand white rabbits (*O. cuniculi*) were purchased from a commercial rabbit farm. All of the rabbits had not been vaccinated with RHDV vaccines, and were of approximately 4 months old, with a body weight of ~1.5 kg. The rabbits were randomly divided into three groups (G1, G2 and G3) with equal numbers per group. Rabbits in the G1 group were inoculated intraperitoneally with 1 mL of 10% 0.22 μm syringe filtrated liver homogenate the same as the initial preparation used for SISPA and sequencing. The G2 group was inoculated subcutaneously with the same dose of the material as the G1 group. G3 rabbits were inoculated intraperitoneally with 1 mL PBS as controls. Rabbits in different groups were housed separately and monitored daily for clinical signs. After death, necropsies were performed immediately, and tissue samples were harvested for histology, immunohistochemistry and RT-PCR detection.

## 3. Results

### 3.1. Clinical and Histopathological Symptoms of Diseased Alpine Musk Deer

**Figure 1:**
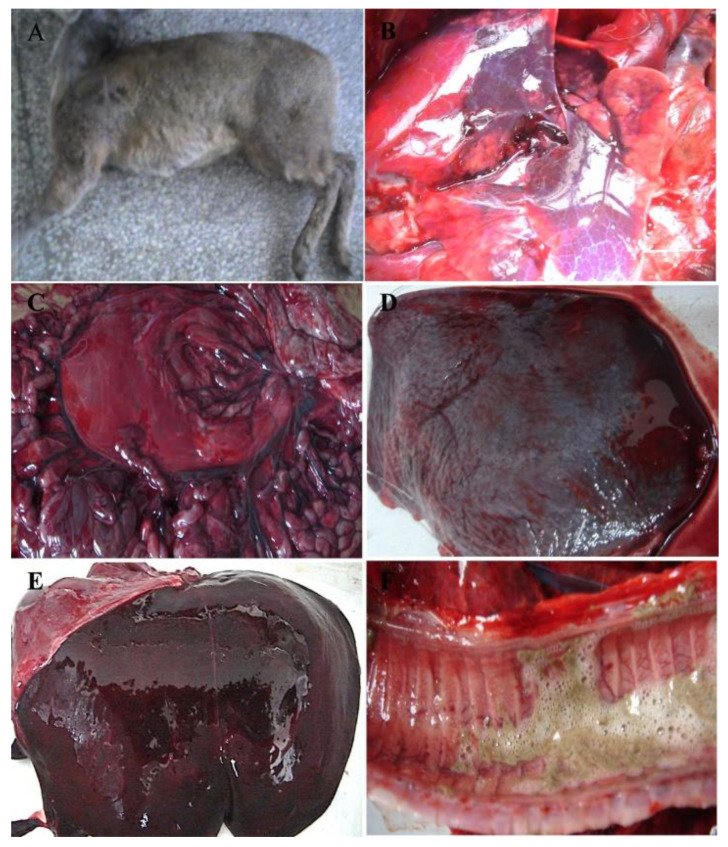
Macroscopic lesions observed in diseased Alpine musk deer. A dead Alpine musk deer (**A**) and common signs of hyperemia, hemorrhage, and necrosis observed in the lung (**B**), stomach and intestine (**C**), spleen (**D**), liver (**E**), and trachea (**F**).

During December 2010 and January 2011, the Alpine musk deer base at Xinglongshan National Natural Reserve suffered sudden deaths of 105 animals from a population of approximately 1000 at that time. Through the investigation, we found that most of the affected deer developed an acute disease and suddenly died without visible clinical signs. The disease mainly occurred in younger animals within one year of age, with a case-fatality rate of approximately 100%. The animals with elongated disease processes showed symptoms of depression, anorexia, and opisthotonos (Figure 1A). Postmortem examination of dead Alpine musk deer showed that hyperemia, hemorrhage, and necrosis were commonly present in most organs. Specifically, the lungs manifested by edematous and congested lobes and patchy areas of consolidation (Figure 1B). The stomach and intestines showed a dark red appearance (Figure 1C). The spleens showed diffuse dark red color, severe hyperemia, and were engorged, with rounded edges (Figure 1D). The livers were enlarged and brittle, showing a diffuse dark red color and severe hyperemia (Figure 1E). The tracheal mucosa was hyperemic and contained abundant yellow frothy fluid (Figure 1F).

**Figure 2:**
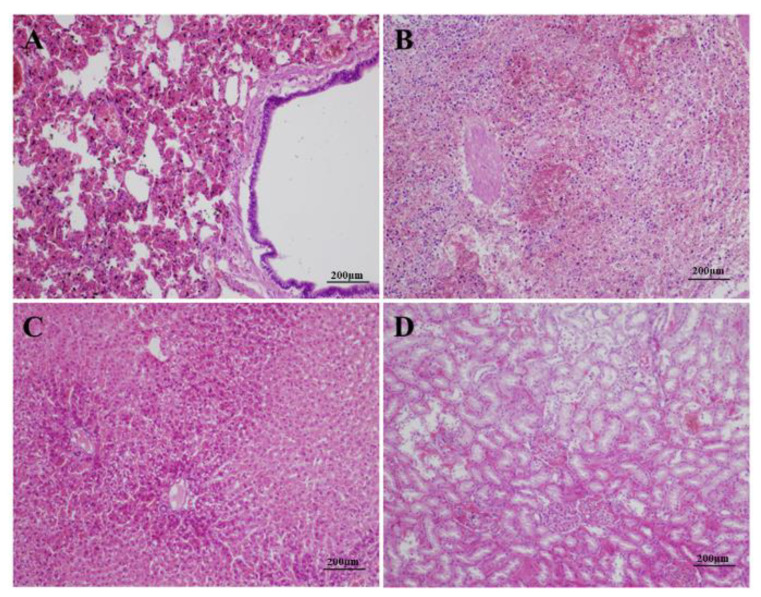
Histopathological lesions observed in diseased Alpine musk deer. Lung (**A**): moderate, diffuse congestion in alveolar capillary and blood vessels, extensive hemosiderin deposition, and dilation of bronchioles; liver (**B**): vacuolar degeneration of hepatocytes in the central zone of the lobule, karyopyknosis and karyolysis in some hepatocytes, and mild sinusoidal dilation and congestion; spleen (**C**): disorganization of the splenic architecture, extensive necrosis and atrophy of the white pulp with severe lymphocyte depletion, and moderate hemorrhage in the parenchyma; kidney (**D**): congestion of interstitial blood vessels, microthrombosis in the glomerular capillaries, and degeneration and karyopyknosis in numerous tubular epithelial cells. (H&E, 100×).

**Figure 3:**
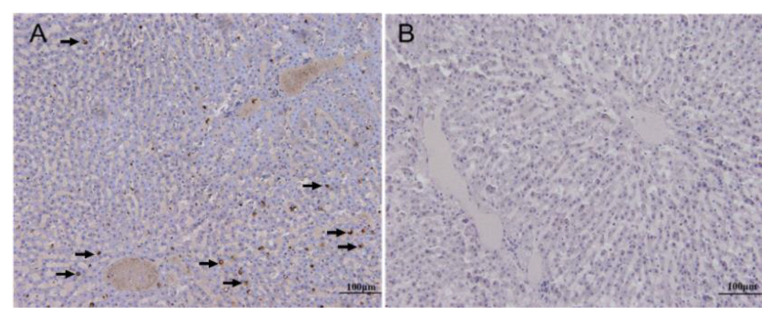
Detection of RHDV VP60 signals in the livers of the dead Alpine musk deer by immunohistochemistry. Incubation with the primary antibodies against VP60 (**A**), positive signals are indicated by arrow; incubation without the primary antibody as the control (**B**).

The histopathological results of the diseased animals showed moderate diffuse congestion in the alveolar capillary and blood vessels, extensive hemosiderin deposition, and dilation of bronchioles in the lung (Figure 2A); disorganization of the splenic architecture, extensive necrosis and atrophy of the white pulp with severe lymphocyte depletion, and moderate hemorrhage in the parenchyma of the spleen (Figure 2B); vacuolar degeneration of hepatocytes in the central zone of the lobule, karyopyknosis and karyolysis in some hepatocytes, and mild sinusoidal dilation and congestion in the liver (Figure 2C); congestion of interstitial blood vessels, microthrombosis in the glomerular capillaries, and degeneration and karyopyknosis in numerous tubular epithelial cells in the kidney (Figure 2D). The results of immunohistochemistry showed that VP60 positive signals, showing as dark brown staining, scattered in the cytoplasm of hepatic cells and sinusoid (Figure 3A). No positive signal was found in the negative control (Figure 3B).

### 3.2. Identification of the RHDV Strain GS/YZ

**Figure 4:**
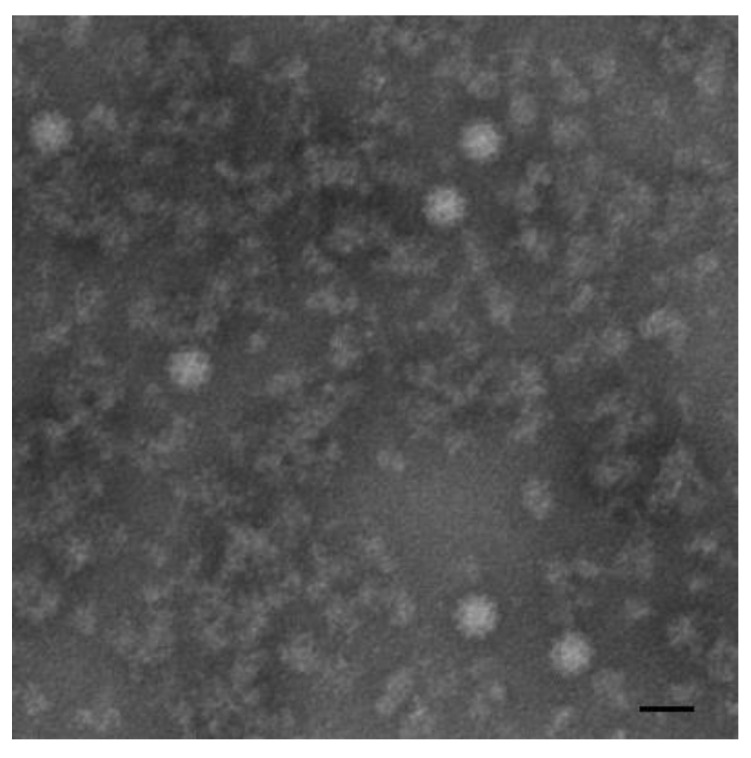
Electron micrograph of calicivirus-like particles from the liver homogenate of diseased Alpine musk deer. Bar: 50 nm.

To identify the potential pathogens, the SISPA method was used to amplify the gene fragments from viral genomes. After the sequencing and analysis of 20 selected positive clones, we obtained 20 gene segments that all showed very high similarities with the genome fragments of RHDV, and no other genes from viruses or bacteria were detected. To detect the potential casual pathogens, negative staining samples were prepared with tissue homogenates collected from the diseased Alpine musk deer and examined by EM. From these samples, we found viral particles ~35 nm in diameter with morphological characteristics of caliciviruses (Figure 4). Taken together, we believe these viral particles are RHDVs and designated the strain as RHDV GS/YZ.

### 3.3. Bacterial Culture and Virus Isolation

The results of the bacterial culture showed that there was no bacterial growth on the enrichment media. No CPE was observed in Vero, HeLa, MDBK, MDCK, CRFK, and RK-13 cells inoculated with the tissue homogenates of dead Alpine musk deer after blind passaging for three generations.

### 3.4. Genomic and Phylogenetic Characteristics of RHDV GS/YZ

To obtain the full-length genome sequence of RHDV GS/YZ, we designed eight pairs of primers, the products of which covered the whole RHDV genome (Table 1). Through amplification and sequencing, we obtained the full-length genome sequence of RHDV GS/YZ, which was 7437 nt in length. The genomic structure was the same as that of other RHDV strains [31], composed of a 5′ noncoding region (nt 1–9), open reading frame ORF1 (nt 10–7044), open reading frame ORF2 (nt 7025–7378), and 3′ noncoding region (nt 7379–7437). To compare the genomewide differences between GS/YZ and other RHDVs, we conducted a multiple sequence alignment with the sequence of GS/YZ and sequences of 85 RHDV strains downloaded from GenBank. The results showed that GS/YZ shared genome sequence identities of 78.7–98.7% with other RHDV strains, the highest of which was with RHDV strain HB (KY437668.1) (Table 2). We also compared the nucleotide and amino acid sequence identities of VP60 of GS/YZ with the reference RHDV strains. The alignment showed that the nucleotide and amino acid sequence identities were 81.9–98.8% and 88.3–99.1%, respectively, with the highest values observed between GS/YZ and the Chinese RHDV isolate WX/China/1984 (AF402614.1) (Table 2), suggesting that GS/YZ is genetically almost identical to the rabbit strains and was probably a “spillover” from rabbits to Alpine musk deer.

**Figure 5:**
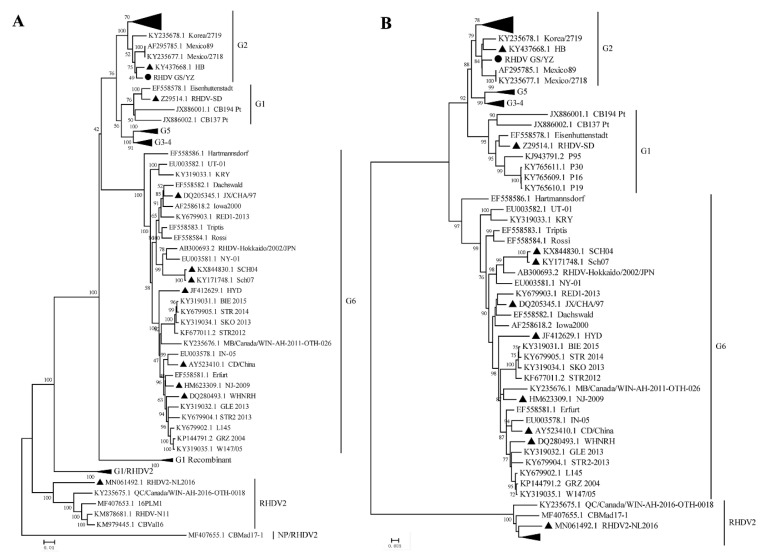
Evolutionary relationships of the new RHDV isolate and reference strains based on the nucleotide sequences of the full-length genome (**A**) and VP60 (**B**). The evolutionary history was inferred using the Neighbor-Joining method implemented in MEGA7 and tested using 1000 bootstrap replicates. Only bootstrap values equal to or greater than 70 are shown. Each sequence is labeled with its GenBank accession number and name beside the branch, with an additional black dot for the new and black triangles for the Chinese strains.

To analyze the phylogeny of GS/YZ, we constructed neighbor-joining trees based on the nucleotide sequences of the whole genome and VP60 of GS/YZ and the reference RHDV strains (*n* = 85). In the whole genome tree (Figure 5A), the new Alpine musk deer strain GS/YZ was clustered within the genogroup G2, showing the closest evolutionary relationships with RHDV strains Chinese HB (KY437668.1), Korea/2719 (KY235678.1), Mexico/2718 (KY235677.1), and Mexico89 (AF295785.1). This phylogeny of the virus was strongly supported by the VP60 tree (Figure 5B), in which the new strain showed almost the same phylogenetic topology as that of the genome tree, indicating that GS/YZ is a G2 RHDV virus, showing close evolutionary relationships with the rabbit isolates.

Given the identity of the new virus isolated from Alpine musk deer, we compared the amino acid differences in VP60, nonstructural proteins, and VP10 of GS/YZ and other G2 RHDV strains (*n* = 19). As shown in Table 3, a total of 21 positions of GS/YZ showed amino acid substitutions compared with those of the HB strain. Seven sites were located in the P2 domain of VP60. Although the amino acids at these positions in GS/YZ were also observed in other strains, the combination of 307Asn + 309Ser + 377Gln was only found in GS/YZ. VP60 is the major capsid protein and is responsible for the attachment and entry of the virus into host cells and determines the host specificity of the virus [32]. Three distinct amino acid residues (p29 150Tyr, RdRp 3Asp, and RdRp 18Asn) were observed in the nonstructural proteins of GS/YZ. In the minor structural protein VP10, only two sites showed variabilities, with the most strains having Val at site 18 in contrast to Ile at that site in GS/YZ. Further studies should be performed to prove whether these specific amino acid substitutions were responsible for the infection of GS/YZ in Alpine musk deer.

### 3.5. Pathogenicity of RHDV GS/YZ in Rabbits

**Figure 6:**
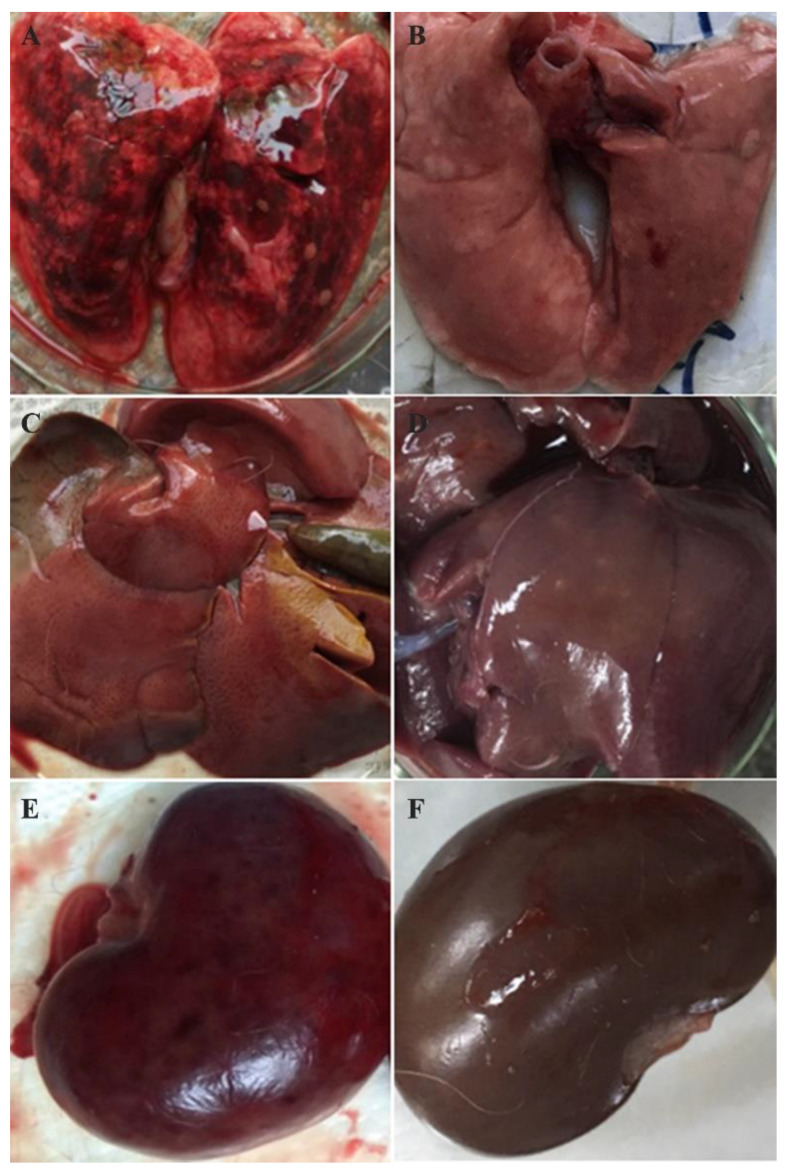
Macroscopic signs observed in rabbits inoculated with the RHDV strain GS/YZ. Lung (**A**): pulmonary hyperemia, and carnification. Liver (**C**): friable, fatty, and discolored liver. Kidney (**E**): necrotic foci on the kidney. The lung (**B**), liver (**D**), and kidney (**F**) of uninfected rabbits.

To evaluate the pathogenicity of RHDV GS/YZ in rabbits, we inoculated six New Zealand white rabbits subcutaneously or intraperitoneally with RHDV GS/YZ-containing tissue homogenates collected from Alpine musk deer. All rabbits died within 72 h postinfection and showed typical clinical signs of RHDV infection in rabbits, including an acute decrease in appetite and activity, depression, opisthotonos, and occlusion of the nares with foam and bloody secretions. Necropsy of the diseased rabbits showed congestion of the tracheal mucosa, pulmonary hyperemia, and carnification (Figure 6A); friable, fatty, and discolored liver (Figure 6C); congestion and enlargement of the spleen, and necrotic foci on the kidney (Figure 6E). No visible changes were observed in the lung (Figure 6B), liver (Figure 6D) and kidney (Figure 6F) of the control rabbits.

**Figure 7:**
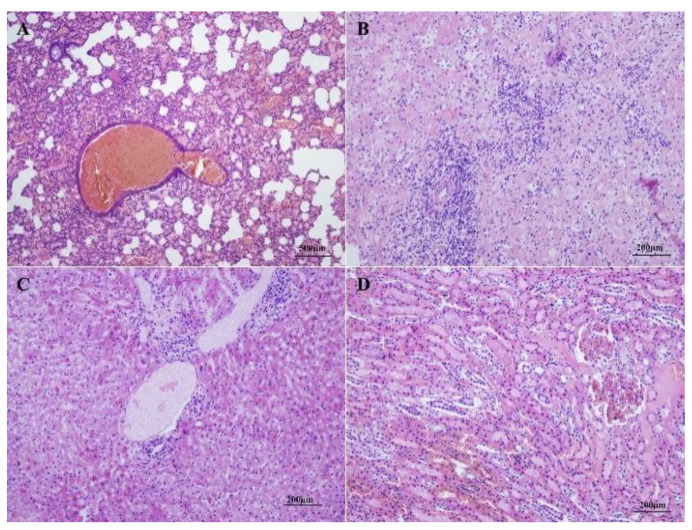
Histopathological changes in rabbits infected with the RHDV strain GS/YZ. Lung (**A**): moderate, diffuse congestion in the alveolar capillary and blood vessels, extensive hemosiderin deposition, and dilation of bronchioles. Liver (**B**): vacuolar degeneration of hepatocytes in the central zone of the lobule, karyopyknosis and karyolysis in some hepatocytes, and mild sinusoidal dilation and congestion. Spleen (**C**): disorganization of the splenic architecture, extensive necrosis and atrophy of the white pulp with severe lymphocyte depletion, and moderate hemorrhage in the parenchyma. Kidney (**D**): congestion of interstitial blood vessels, microthrombosis in the glomerular capillaries, and degeneration and karyopyknosis in numerous tubular epithelial cells [33,34].

**Figure 8:**
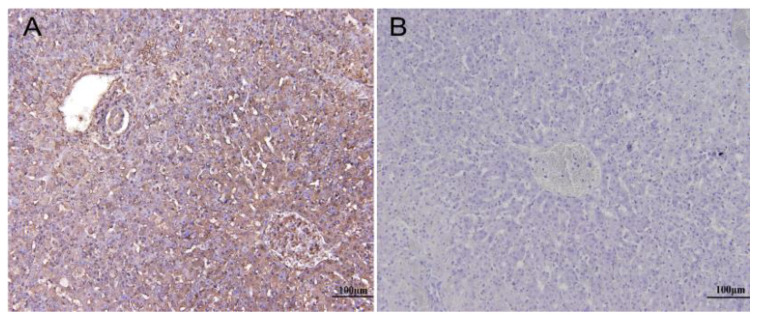
Detection of RHDV VP60 signals in the livers of the rabbits inoculated with the tissue homogenates of the dead Alpine musk deer by immunohistochemistry. Incubation with the primary antibodies against VP60 (**A**); incubation without the primary antibody as the control (**B**).

Histopathologically, rabbits of both inoculated groups showed similar lesions. In the lungs, extensive and diffuse alveolar edema and moderate congestion of small blood vessels were observed (Figure 7A). The livers were manifested with severe, multifocal necrosis, indicated by mild to moderate inflammatory infiltrate and consisted of lymphocytes in portal spaces, scattered intralobular hemorrhagic foci, and single or groups of hepatocytes showed acidophilic shrinkage (Figure 7B). Similar to those of RHDV-infected Alpine musk deer, the spleens of the rabbits showed disorganization of the splenic architecture and extensive necrosis and atrophy of the white pulp with severe lymphocyte depletion (Figure 7C). In the kidneys, microthrombosis presented in the glomerular capillaries, numerous tubular epithelial cells showed degeneration and karyopyknosis, the tubular lumen was dilated and filled with casts, and focal hemorrhages were also observed (Figure 7D). The immunohistochemistry results showed that very strong VP60-positive signals with diffuse and intense dark brown staining were distributed broadly in the cytoplasm of the hepatic cells and sinusoids of the rabbits (Figure 8A), while no signal was observed in the controls (Figure 8B).

In addition, RHDV genes were detected in all liver samples of the infected rabbits by using RT-PCR methods. These results indicate that GS/YZ caused typical macro- and microscopic lesions in rabbits similar to that of other RHDVs.

## 4. Discussion

The Alpine musk deer has been listed as an endangered animal species. Protecting them from fatal diseases has priority over other protective actions in China. The fatal disease that occurred in Xinglongshan National Natural Reserve has posed a severe threat to the Alpine musk deer population. The identification of potential causative agents of the disease is of great importance for their protection. During the causative agent investigation process, we identified, for the first time, an RHDV strain from the diseased Alpine musk deer. Although the causative agent of this disease has not been determined yet, the isolation of the RHDV from the diseased deer provided an important clue and direction for further studies.

The clinical manifestations presented in the diseased Alpine musk deer indicated that it was an acute, hemorrhagic disease. In peracute cases, the animals died very quickly without visible clinical signs, while in acute cases, infected animals showed anorexia, apathy, and opisthotonos. Hemorrhages, congestions, and necrosis could be observed in several organs, particularly in the lungs, livers, spleens, and kidneys (Figure 1 and Figure 2). All of these macroscopic and microscopic lesions in the Alpine musk deer were similar to that presented in RHDV-infected rabbits (Figure 7 and Figure 8). The immunohistochemical results showed that the RHDV antigen could be detected in the livers of dead Alpine musk deer using the RHDV VP60 specific antibodies (Figure 3), which indicated that the dead Alpine musk deer were indeed infected by RHDV. However, because Alpine musk deer are protected by the wildlife protection laws of China, we could not conduct the RHDV infection experiment in Alpine musk deer and have not obtained the direct evidence to support that the fatal disease in the Alpine musk deer had been caused by RHDV infections. Therefore, the causative agent of this disease in the deer population is still to be determined.

During the investigation, we obtained epidemiological data on the outbreak, and a total of 105 animals had died by the time we collected the data. However, there were only three deer available for us to conduct the clinical examination and collect samples. Therefore, we conducted the following experiments based on the specimens collected from the three deer. This limited our efforts to obtain sufficient evidence to show that the disease was caused by RHDV. After obtaining the results for RHDV infection in the Alpine musk deer, we tried to conduct a serological investigation in the deer population. However, the deer are timid and easily frightened, and they were difficult to capture. Therefore, we have not yet obtained serological evidence. However, we will continue our efforts to obtain more knowledge about this disease.

The identification of a causative agent in an emergent infection is a great challenge, especially in one that is caused by a novel pathogen. EM is suitable for detecting pathogens in such situations [35,36]. In this study, we identified calicivirus-like particles in the liver homogenates of the dead Alpine musk deer by EM, which provided an important clue for ultimately identifying RHDV GS/YZ. Regrettably, we could not perform direct EM examination on the liver cells of the deer. We did not collect specimens for preparing ultrathin sections. Another technique especially suitable for detecting novel viruses is SISPA [26]. Based on the SISPA results, all 20 clones selected for sequencing were RHDV-positive, and no other viral or bacterial genes were detected, which strongly supported the EM findings. These consistent results indicated that we identified an RHDV stain from the diseased Alpine musk deer.

The genome characteristics and phylogeny of Alpine musk deer RHDV GS/YZ indicated that this virus is a G2 RHDV, showing close genetic and evolutionary relationships with previously reported G2 RHDV isolates (Figure 5). To date, all other RHDV strains in the G2 group were isolated from rabbits, and no spillover of these viruses to other animal populations was found [37,38,39]. To understand the potential reasons for this spillover, we compared the amino acid differences of RHDV GS/YZ and 19 other G2 RHDV strains. We found a total of 21 variable sites that were located in VP60 (*n* = 7), nonstructural proteins (*n* = 12), and VP10 (*n* = 2), respectively (Table 3). The unique substitutions (S307N + A309S + E377Q) were found in the VP60 of GS/YZ. The P2 domain of VP60 is responsible for receptor binding and determines the host specificity of RHDV [40,41,42]. A previous study showed that a specific integrin recognition motif was produced by changing two amino acids (S305R and N307D) in VP60 [41]. Therefore, the substitutions in the VP60 of GS/YZ might affect the receptor binding property of the virus. RdRp is responsible for replicating the viral genome and may also be responsible for differences in virulence [43,44]. We found two distinct amino acids in the RdRp of GS/YZ, and these changes may influence the performance of the virus in terms of viral genome replication and virulence. RHDVs are believed to replicate strictly only in lagomorphs [23]. In addition to our findings, there were also other reports showing that both RHDV-specific antibodies and viral RNA have been found in other animals [24,25,26], suggesting that spillover of RHDV in other animal species probably happened, but the underline molecular basis for this spillover needs to be explored in the future.

As we mentioned above, RHDV experimental infection cannot be performed in Alpine musk deer, this limited us to replicate the disease in the Alpine musk deer. Given the nonculturable nature of RHDV, we verified the isolation of RHDV strain GS/YZ by inoculating rabbits with liver homogenate collected from the diseased Alpine musk deer. Typical clinical signs, pathological and histopathological alterations, and mortality rates of RHD were reproduced in the rabbits, suggesting that the livers of the dead deer contained large amounts of the virus, and this virus was similar in virulence to that of other rabbit-origin RHDV strains in rabbits. This was further supported by the immunohistochemistry and RT-PCR assays, which showed that both antigens and genes from RHDV were present in the liver cells of the rabbits.

In the present study, we used liver homogenate instead of purified viral particles in the inoculation experiments in rabbits, which was based partially on the evidence that showed that no other virus or bacteria was detected in the homogenate as well as protocols from previous studies. In the previous studies, rabbits inoculated with liver homogenate typically developed the clinical and histopathological characteristics of RHDV infection, which was consistent with our results. The immunohistochemistry and RT-PCR results of our study also proved that RHDV was the only pathogen present in the livers of the infected rabbits, suggesting that the animal infection experiment was successful. In order to provide more convincing evidence, further challenging experiments with liver homogenate collected from the diseased Alpine musk deer in immunized rabbits with the RHDV vaccine can be performed in the future. If the rabbits do not develop clinical syndromes, this will indirectly confirm that the RHDV is the dominating pathogen that caused the outbreak in Alpine musk deer.

In conclusion, we successfully isolated an RHDV strain from dead Alpine musk deer with acute hemorrhagic disease. This virus belongs to the genogroup G2 of RHDV and is highly pathogenic in rabbits. Our findings extended the epidemiology and probably the host range of RHDV.

## Figures and Tables

**Table 1:** Primers designed for detecting and amplifying the complete genome of rabbit hemorrhagic disease virus (RHDV) strain GS/YZ.

Primer	Location	Sequence (5′–3′)	Length (bp)
K9N		GACCATCTAGCGACCTCCCANNNNNNNNN	
K		GACCATCTAGCGACCTCCCA	
RHDV-F1	1–30	GTGAAAGTTATGGYGGCTATGTCRCGCCTT	1363
RHDV-R1	1333–1363	CCTCRTTRGCCATTTTCACAACTGTCATAAC	
RHDV-F2	1222–1244	GGTGCTGGCAARCTCACAACCTT	1097
RHDV-R2	2290–2318	CCYTCGATATGTGAACTTGATTGTATGGG	
RHDV-F3	2224–2255	TGGAARCAGTACTTTGTYATGTATGGTTGTGT	1375
RHDV-R3	3569–3598	CAATCTTKGTGGAGTCTGACAATGTCTTCT	
RHDV-F4	3296–3322	ATGYTCWCCCACYACTGAYCTGTGCCT	1156
RHDV-R4	4426–4451	GRGATGTCATRTCAACGCCAACTGC	
RHDV-F5	4352–4328	TGYTRTGGGGYTGTGACGTTGGTGT	971
RHDV-R5	5298–5322	CGGGCTTTGCCCTCCATAACATTC	
RHDV-F6	5209–5240	CARTTYARTGTTTACAGCTACGATGCTGCTAG	1499
RHDV-R6	6678–6707	TGACRACAGAYGCRAACATGATGGGTGTG	
RHDV-F7	6377–6402	GTGCAATCTGGAACAGTAACAGCGGT	995
RHDV-R7	7335–7371	CTGGAYTCRCCWGTGGTRTTATARATCTTAACACTAT	
3′-terminus-F	6942–6967	CTTGACTGAACTCATTGACGTACGCC	536
TX22	7372–7394	GGGAATTCCATATGTTTTTTTTTTTTTTTTTTTTTT	

**Table 2:** Identities of nucleotide and deduced amino acid sequences of RHDV GS/YZ with 18 selected reference RHDV strains.

RHDV Strain	GenBank Accession No.	Country	Genotype and Genogroup	RHDV GS YZ						
				Nucleotides (%)				Amino Acids (%)		
				Genome	NSP *	VP60	ORF2	NSP *	VP60	VP10
RHDV-SD	Z29514.1	China	G1	95.0	94.6	96.1	96.6	98.0	98.1	96.6
Hartmannsdorf	EF558586.1	Germany	G1	90.9	89.3	95.2	96.3	96.1	96.6	97.5
CB137 Pt	JX886002.1	Portugal	G1	93.0	93.6	94.3	94.4	97.8	96.6	94.1
HB	KY437668.1	China	G2	98.7	98.8	98.5	98.6	99.3	98.6	98.3
WXChina 1984	AF402614.1	China	G2	-	-	98.8	-	-	99.1	-
Korea/2719	KY235678.1	Korea	G2	97.9	97.7	98.3	98.6	99.1	98.8	97.5
Mexico/2718	KY235677.1	Mexico	G2	98.5	98.5	98.4	98.9	99.3	98.8	99.2
Lagovirus europaeus/GI. 1d/O cun/FR/2000/00-21	MH190418.1	France	G5	94.0	93.4	95.7	95.0	97.6	97.8	93.9
Jena	EF558576.1	Germany	G5	94.9	94.4	96.4	97.2	97.8	97.6	94.9
Sch07	KY171748.1	China	G6	89.7	88.4	92.1	95.5	96.2	95.2	96.6
CD/China	AY523410.1	China	G6	89.9	88.5	92.7	96.0	96.3	95.3	97.5
WHNRH	DQ280493.1	China	G6	89.9	88.5	92.6	94.4	95.9	95.9	96.6
NJ-2009	HM623309.1	China	G6	90.3	88.9	93.2	95.5	96.7	95.9	97.5
NY-01	EU003581.1	USA	G6	89.8	88.3	93.3	96.0	95.9	96.2	97.5
STR2012	KF677011.2	Poland	G6	89.9	88.6	92.9	95.2	96.4	96.0	98.3
RHDV2-NL2016	MN061492.1	China	RHDV2	85.1	86.7	82.2	86.2	95.8	88.4	83.1
CBMad17-1	MF407655.1	Portugal	RHDV2	78.7	77.3	82.5	84.5	88.2	88.4	83.3
AUS/NSW/BRO-2/2015	MF421648.1	Australia	RHDV2	87.9	90.2	81.9	85.7	97.2	88.3	83.3

*: Nonstructural protein. -: no sequence.

**Table 3:** The amino acid differences of VP60, nonstructural proteins, and VP10 of the RHDV GS/YZ with the G2 strains.

Viruses	Amino Acid Sites																				
	VP60							p16		p23	p29			Pro	RdRp					VP10	
	299	307	309	377	386	412	426	51	138	57	51	138	150	8	3	18	148	478	491	18	84
HB	K	G	A	E	N	T	V	I	S	N	R	K	A	Y	N	H	S	H	D	V	T
GS/YZ	R	N	S	Q	G	A	I	N	P	S	K	R	T	H	D	N	A	Q	A	I	A
Mexico/2718	R	S	A	E	G	A	I	N	P	N	R	R	A	H	N	H	A	Q	A	V	A
Mexico89	R	S	A	E	G	A	I	N	P	N	R	R	A	H	N	H	A	Q	A	V	A
Korea/2719	R	S	A	E	G	T	I	N	P	N	R	K	A	H	N	H	A	Q	A	V	A
PD_1989	R	S	A	E	G	A	I	N	P	N	R	K	A	H	N	H	A	Q	A	V	A
KGM_1988	R	S	A	E	G	A	I	N	P	S	R	K	A	H	N	H	A	Q	A	V	A
MAL	R	S	A	E	G	A	I	N	P	N	R	K	A	H	N	H	A	Q	A	V	A
FRG	R	S	A	E	G	A	I	N	P	N	R	K	A	H	N	H	A	Q	A	V	A
Italy-90	R	S	A	E	G	A	I	N	P	N	R	K	A	H	N	H	A	Q	A	V	A
RHDV-V351	K	S	A	E	G	A	I	N	P	N	R	K	A	H	N	H	A	Q	A	V	A
NZ54	R	S	A	E	G	A	I	N	P	N	R	K	A	H	N	H	A	Q	A	V	A
NZ61	R	S	A	E	G	A	I	N	P	N	R	K	A	H	N	H	A	Q	A	V	A
AUS/ACT/AIN-1/2009	R	S	A	E	S	N	I	N	P	S	K	K	A	H	N	H	A	Q	A	V	A
AUS/ACT/GUN1-52/2009	R	G	A	E	S	N	I	N	P	S	K	K	A	H	N	H	A	Q	A	V	A
AUS/ACT/MtPt-4/2010	R	S	A	E	S	N	I	N	P	S	K	K	A	H	N	H	A	Q	A	V	A
AUS/ACT/PI-1/2009	R	S	A	E	S	N	I	N	P	S	K	K	A	H	N	H	A	Q	A	V	A
AUS/NSW/M9/2007	R	S	A	E	S	N	I	N	P	S	K	K	A	H	N	H	A	Q	A	V	A
AUS/SA/ORA383/2008	K	N	A	E	G	N	I	N	P	N	R	K	A	H	N	H	A	Q	A	I	A
AUS/WA/B.Hill/2013	K	N	A	Q	G	N	I	N	P	S	R	K	A	H	N	H	A	Q	A	I	A
